# Current Status and Future Trends in Removal, Control, and Mitigation of Algae Food Safety Risks for Human Consumption

**DOI:** 10.3390/molecules27196633

**Published:** 2022-10-06

**Authors:** Guowei Wu, Dingling Zhuang, Kit Wayne Chew, Tau Chuan Ling, Kuan Shiong Khoo, Dong Van Quyen, Shuying Feng, Pau Loke Show

**Affiliations:** 1 Department of Chemical and Environmental Engineering, Faculty of Science and Engineering, University of Nottingham Malaysia, Semenyih 43500, Malaysia; ebxgw1@nottingham.edu.my; 2 Institute of Biological Sciences, Faculty of Science, Universiti Malaya, Kuala Lumpur 50603, Malaysia; s2032669@siswa.um.edu.my (D.Z.); tcling@um.edu.my (T.C.L.); 3 School of Chemistry, Chemical Engineering and Biotechnology, Nanyang Technological University, 62 Nanyang Drive, Singapore 637459, Singapore; 4 Department of Chemical Engineering and Materials Science, Yuan Ze University, Taoyuan 32003, Taiwan; kuanshiong.khoo@hotmail.com; 5 Institute of Biotechnology, Vietnam Academy of Science and Technology (VAST), Hanoi 100803, Vietnam; dvquyen@ibt.ac.vn; 6 Vietnam Academy of Science and Technology, University of Science and Technology of Hanoi, Hanoi 100803, Vietnam; 7 Medical College, Henan University of Chinese Medicine, Zhengzhou 450046, China; 8 Department of Sustainable Engineering, Saveetha School of Engineering, SIMATS, Chennai 602105, India; 9 Zhejiang Provincial Key Laboratory for Subtropical Water Environment and Marine Biological Resources Protection, Wenzhou University, Wenzhou 325035, China

**Keywords:** algal food, food safety, foodborne diseases, microbial foodborne pathogens, farm-to-table chain

## Abstract

With the rapid development of the economy and productivity, an increasing number of citizens are not only concerned about the nutritional value of algae as a potential new food resource but are also, in particular, paying more attention to the safety of its consumption. Many studies and reports pointed out that analyzing and solving seaweed food safety issues requires holistic and systematic consideration. The three main factors that have been found to affect the food safety of algal are physical, chemical, and microbiological hazards. At the same time, although food safety awareness among food producers and consumers has increased, foodborne diseases caused by algal food safety incidents occur frequently. It threatens the health and lives of consumers and may cause irreversible harm if treatment is not done promptly. A series of studies have also proved the idea that microbial contamination of algae is the main cause of this problem. Therefore, the rapid and efficient detection of toxic and pathogenic microbial contamination in algal products is an urgent issue that needs to be addressed. At the same time, two other factors, such as physical and chemical hazards, cannot be ignored. Nowadays, the detection techniques are mainly focused on three major hazards in traditional methods. However, especially for food microorganisms, the use of traditional microbiological control techniques is time-consuming and has limitations in terms of accuracy. In recent years, these two evaluations of microbial foodborne pathogens monitoring in the farm-to-table chain have shown more importance, especially during the COVID-19 pandemic. Meanwhile, there are also many new developments in the monitoring of heavy metals, algal toxins, and other pollutants. In the future, algal food safety risk assessment will not only focus on convenient, rapid, low-cost and high-accuracy detection but also be connected with some novel technologies, such as the Internet of Things (artificial intelligence, machine learning), biosensor, and molecular biology, to reach the purpose of simultaneous detection.

## 1. Introduction

Algae are among the most common organisms on earth and are defined as a class of autotrophic plants with no differentiation of roots, stems, and leaves; no vascular bundles; and containing photosynthetic pigments in terms of biology [1]. Most of them exist in aquatic environments, but a few live in terrestrial habitats, meanwhile they can grow in both fresh and saltwater [2]. For example, the well-known *Chlorella*, *Spirogyra*, *Chlamydomonas*, and *Pandorina* are grown in freshwater. On the contrary, marine algae, such as diatoms, brown algae, green algae, and red algae, grow in seawater. Previous studies have proved that algae are able to survive in extreme conditions [3,4]. Guiry reported in 2012 that estimates of the total number of algal species ranged widely, from 30,000 to over 1 million. He also mentioned a seminar presentation that suggested an extreme estimate of 350 million species, though he did not endorse this figure. Using the online taxonomic database AlgaeBase, he arrived at a much more conservative estimate of approximately 72,500 species [5]. As a result, the population of algae is quite enormous. Based on this reason, the study of the value and application of the most abundant algae has attracted a large number of researchers. On account of the urgent demand for renewable and clean energy in human society, the current research scope on algae mainly focuses on seaweed plants as a renewable and sustainable energy source. For instance, some researchers and research institutions have used microalgae as biomass to produce renewable and sustainable energy. As early as 2014, Kim changed the concentration of carbohydrates in the endophytic substances of microalgae by adopting the method of nutrient stress culture, and 89% of hydrolysates of microalgae could be successfully converted into ethanol fuel under the condition of continuous immobilized yeast fermentation [6]. At that time, using microalgae as a carbon source raw material to produce bioethanol became one of the hotspots of this field. Meanwhile, microalgae are also known as the third generation of bioethanol different from traditional energy sources. Furthermore, biodiesel has been produced from the extraction of lipid-rich algae, such as *Chlorella*, *Selenoses*, and *Dinoflagellates*, over the past decade and methods of extraction have also been improved [7,8,9,10]. In addition to the development of new liquid fuels, research on solid fuels is not standing still. Because microalgae are excellent biomass feedstock, which have advantages, including high calorific value and clean and environmental properties. Therefore, it is processed into biochar by pyrolysis, drying, and carbonization to replace traditional coal [11]. Unlike the above use of microalgae as biomass to produce biodiesel and bioethanol, Chia (2022) proposed that using algae-based microbial fuel cells has great potential to replace current non-renewable fuels (oil, coal) and solve the current international carbon neutrality problems (climate change, environmental pollution, energy shortage), and the cost of production is lower than that of microalgae biodiesel and bioethanol [12].

Regarding the classification of seaweed products, in general, edible seaweeds can be broadly divided into three categories: red, green, and brown algae [13]. To address the problem of hunger on a global scale, governments and organizations need to take immediate action to transform agri-food systems if they are to meet their commitment to end hunger by 2030 [14]. Therefore, algae are particularly important as a sustainable edible resource [15]. Along with the rapid development of the economy and productivity, people in many countries pay more attention to their own diet health and nutrition. As a result, algae food is favored by consumers because of its high nutrient content. Numerous researchers have shown that seaweeds are rich in proteins, carbohydrates, lipids, and polyunsaturated fatty acids (PUFAs) [16,17]. It is interesting to note that the quality of algal protein is better than that of other plant sources, including wheat, beans, or rice [18]. Moreover, seaweeds contain various vitamins: A, B_1_, B_12_, C, D, and E, riboflavin, niacin, pantothenic acid, and folic acid, etc. [19]. Algae are also rich in trace elements and minerals [20]. In addition, some recent researchers have found that it also has dietary fiber [21,22,23,24], known as the seventh nutrient. Hence, seaweeds are increasingly important as a food resource and are made into medicine and health care products. At the same time, current biologists and chemists pay particular attention to bioactive substances and functional mechanisms of algae.

The positive impact of economic benefit on social development shows an upper trend, which is in sync with consumers also paying more and more attention to their health. Despite edible algae contain plenty of vitamins, micronutrients, and many plant compounds, according to the relevant research reports from Europe, South America, and Asia, the algae food safety situation demonstrates that consumers have to face the problems caused by food safety incidents [25,26,27]. Stewart (2008) found that there is no direct evidence of toxic effects from the consumption of astaxanthin-rich microalgae in rats through acute and subchronic toxicity studies, but it needs to pay attention to the amount of intake [28]. As early as 2013, the European Commission formulated relevant laws and regulations based on an extensive analysis of the potential hazards of seaweed products [29]. However, after years of changes in technological development and the transformation of industry 4.0 to 5.0 in algae [30], some parts of laws and regulations are out of date. Therefore, it is urgent to strengthen the awareness of algae food safety and take measures to protect consumers’ health.

This review aims to integrate the three main factors, including physical, chemical, and biological hazards, that affect the food safety of algae in detail. It also points out the effects of these factors on human health, respectively. In addition, based on numerous studies on toxic and hazardous substances in algal food, we analyze the current situation of seaweed food safety testing technology, especially concerning heavy metals and algal toxins. By comparing the advantages and limitations of poison detection technology, we forecast the development trend of seaweed food safety testing, so that it provides a basis for further research in the field of algae food safety.

## 2. Classification of Algal Food and Its Application in Food Industry

### 2.1. Classification of Algal Food

**Figure 1:**
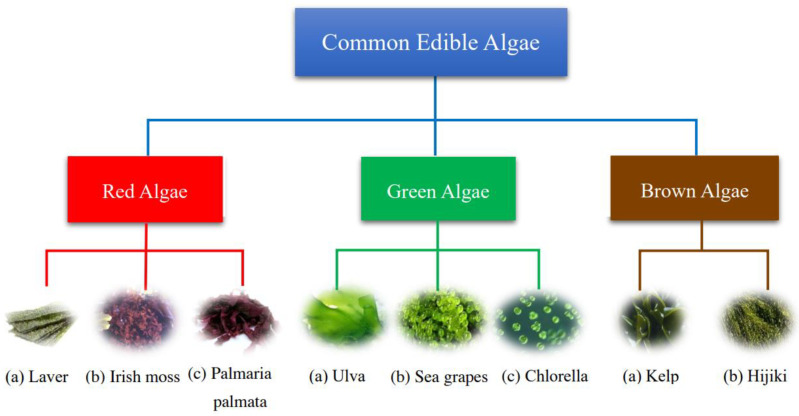
Classification and designation of common edible algae.

Regarding the classification of algae, the Irish botanist and algologist William Henry Harvey first proposed in 1836 to divide known algae into four categories, according to the color of their thallus [31]. Over the past three centuries, along with the development of science, the classification method became a comprehensive system. At present, the widely accepted method of classification is broadly divided into two types. The first classification method is to classify algae into macroalgae and microalgae, according to their size. Another classification is to distinguish the pigment contained in the algal cells, as well as reserved metabolites and cell wall composition. All edible algae known to humans can be classified into these three different types by color (Figure 1). Regarding red seaweeds, laver is the one of most common edible red algae in daily life because of a great deal of people’s consumption. According to biological classification, it belongs to the genus of *porphyra*. In addition, laver is particularly popular in Asian countries, especially in China, Japan, and South Korea. This is due to seaweed not only being able to be eaten directly by drying, baking, and souping [32] but also because consumers can use laver as a raw material to make other foods, such as sushi and rice ball. On the other hand, there are different eating habits of Asian people; some citizens who live in Western countries have a particular fondness for Irish algae (Irish moss) and *Palmaria palmata* (dulse) as their food and food supplement. According to a study in Europe in 2021, the researchers found that dulse is also widely used locally as a heavy source of food and medicine [33].

In terms of green algae, the most widespread is named Ulva, it can be found almost everywhere from Alaska to South Korea. It has been used in salads and soups in Scotland and Ireland in recent years. However, it is commonly used as a condiment in East Asia now [34]. Up to now, several studies have shown that it contains a lot of amino acids, vitamin E, fatty acids, and dietary fiber nutrients, so consumers now pay abundant attention to the product and not just as a seasoner [34,35,36,37]. Second, sea grapes are a general term for edible species of green seaweed belonging to the genus of *Caulerpa* [38]. It grows mainly in the Indo-Pacific region and has a slightly salty taste [39]. Finally, *Chlorella* is a very famous green algae group in microalgae, due to it being rich in proteins, lipid polysaccharides, carotenoids, and other active substances, known as the new resources of edible algae in the future [40,41].

With regard to brown seaweeds, they are called brown algae because their color depends on the ratio of the brown pigment (fucoxanthin) to the green pigment (chlorophyll) [42]. Two of the most commercially important categories are kelp and hijiki, though they belong to different taxonomic groups. “Kelp” is a general term often used to describe species within the order Laminariales, particularly the family Laminariaceae. While historically linked to the genus *Laminaria*, commercial kelp also encompasses the genus *Saccharina* [43], as taxonomic revisions have reclassified several species from *Laminaria* to *Saccharina*. For instance, sugar kelp (*Saccharina latissima*) is renowned for its high nutritional value, containing significant amounts of protein, carbohydrates, vitamins, amino acids, and minerals [44]. Recent studies have shown the regulatory effect of polysaccharides in hijiki on the intestinal flora. Its ethanol extract can regulate intestinal flora and metabolites in patients with type II diabetes so that it can reduce high-fat diet/Streptozotocin (HFD/STZ) to induce hyperglycemia [45,46].

### 2.2. Algal Application in Food Industry

In meat-processing plants, algal extracts such as hydrocolloids are widely applied as food additives to improve texture and sensory attributes [47]. With the advent of artificial meat, algae with high protein content have become a novel source of high-quality protein. Most of this plant protein is microalgae, such as *Spirulina*, *Chlorella*, etc. They are widely used by researchers to develop meat substitutes [48,49]. Currently, nutritionists confirm that *Spirulina* is an excellent source of natural protein food, and it contains up to 60–70% protein content, where human absorption rate are up to 95% [50,51]. This is great news for vegetarians and researchers developing alternative plant proteins. In the beverage industry, algae provide an enormous supply of bioactive ingredients, and lots of studies use different types of algae to make functional drinks. As early as 2003, Takeshi Nagai and Takakiyo Yukimoto successfully made drinks from four different kinds of seaweed and tested their anti-oxidation function. The results showed that these drinks had strong antioxidant activity [52]. Now a new study shows the novel trend of a drink made from seaweeds have the amount of untapped potential that can protect human health [53]. Comparing edible seaweeds with commercially available dairy products, the calcium of cheese cannot be absorbed by consumers who lack enzymes to digest casein. However, those people can absorb calcium directly from algal foods. Therefore, the calcium of seaweeds is better than that of dairy products from the perspective of biological absorption and utilization [54]. Additionally, while dairy products are the main recognized dietary source of calcium, and the average calcium content of dairy products is 100 mg/100 g [55], compared with the calcium level of seaweeds, such as the calcium of the red seaweed *Lithothamnion*, which accounts for 31% of its weight [56], the content is significantly higher than the average calcium content of dairy products.

## 3. Physical Factors Affecting Food Safety in Algae

### 3.1. External Matter in Food Processing

Although many detection techniques and equipment are used on the production line, foreign bodies are still not completely avoided in the algae food processing process. The main reason for this issue is that some employees do not comply with the personnel hygiene requirements of Good Manufacturing Practices (GMP) (e.g., 21 CFR 117.10), leading to the potential inclusion of physical contaminants such as jewelry, hair, plastics, or other foreign materials [25,57]. Meanwhile, due to the lack of regular maintenance of the equipment of food processing enterprises, parts of the equipment will appear in the production of seaweeds. As a result, the food manufacturing factory should obey the GMP and HACCP principles to avoid such problems [58,59].

Apart from the problems that may occur on the food production line, there are foreign bodies in the seaweed products due to the contamination of raw materials. With some news reports in recent years, plastic pollution in the ocean has become a threat that people have to face. Although there is not sufficient evidence for the presence of microplastics in seaweeds currently [60], Lars Gutow et al. reported (2016) that microplastics attached to the surface of algae tend to migrate further into the algae [61]. This has caused concern among consumers about the physical hazards of these foreign matters in marine food. Therefore, the method to monitor and control plastic pollution in the ocean and minimize the harm to seaweed and aquatic environment has become a focus on the physical hazards of algae food in current society.

### 3.2. Radioactive Contamination

**Figure 2:**
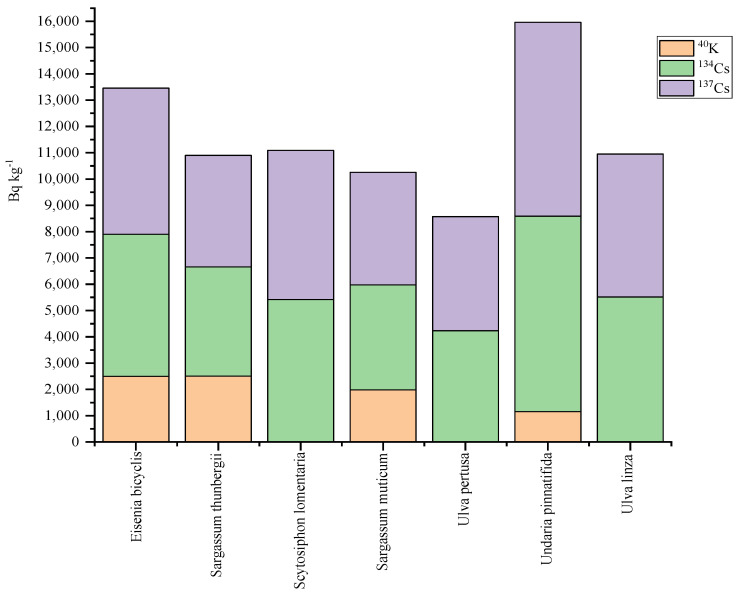
Distribution of three types of radioactive substances accumulated in seven different species of algae in the Fukushima Shioyazaki nuclear accident [62].

Since a large number of edible algae live in the ocean, it is exposed to radioactive pollution and plastic pollution, especially radiation from radioactive pollutants. Mayumi Yoshimura and Akio Akama studied the effects of Japan’s Fukushima nuclear power plant accident on marine algae in 2014. The work highlighted the need for monitoring and management of radioactive contaminants in various sources including algae, to reduce the radioactive cesium values in fish [62]. Similarly, Hiroshi Kawai et al. also investigated the radioactive substance, cesium, that accumulates to seaweeds, which was caused by the nuclear power plant leakage accident in the same year [62]. In the early stage of the impact of nuclear leakage on algae (May 2011), the Cs and Cs content measured in most frozen algae samples were higher than 3000 Bq kg^−1^, with the maximum content reaching 7433.50 and 7371.20 Bq kg^−1^, respectively, indicating that the nuclear pollution to algae was the most serious in the early stage of radiation. Comparing the measurements taken in May with those taken in July, the amount of radioactive cesium in algae in the ocean is falling quite fast. This may be due to algae blooms in the ocean during June and July; meanwhile, the algae that absorb large amounts of radiation die off. Some studies have reported that radiocesium, particularly ^137^Cs with a physical half-life of approximately 30 years, is the primary long-term radionuclide of concern released from Fukushima, affecting forest, freshwater, and coastal marine ecosystems [62,63,64]. In order to investigate the effects of nuclear radiation residues, eight Japanese researchers measured radionuclide levels of 15 seaweed species collected from contaminated areas of Fukushima between May 2012 and June 2015 and considered ecological analyses [65]. According to their idea of comparing cesium ratios of different nuclides, the data measured by Kawai et al. in 2014 [62] were conducted graphic analysis (Figure 2) and it was found that Undaria Pinnatifida had the most types and the highest concentration of nuclear radiation.

After the nuclear disaster happened 10 years ago, the Japanese government announced the release of contaminated water from Fukushima into the sea in April 2021. This decision has caused panic among many consumers around the world, especially those who buy algae-based food. Consumers believe that the radioactive residues of radiation can accumulate in their bodies because humans were at the peak point of the food chain and bioaccumulation [66,67]. Furthermore, the customs of various countries prohibited the entry of seafood, especially algae, exported from Japan [68]. According to a study by Desideri et al. [69], human consumption of algae food contains some different concentrations of radiochemical elements. Therefore, seaweed needs to be monitored more closely to prevent radioactive contamination.

## 4. Chemical Factors Affecting Food Safety in Algae

### 4.1. Iodine

The amount of iodine in foods varies; seaweeds as a marine source have the highest iodine [70]. In terms of nutrition, iodine is an essential trace element for the human body. It is involved in the synthesis of thyroid hormones T3 and T4 and is the main raw material for the body to synthesize thyroid hormones [71]. It is responsible for the development of the central nervous system and participates in and regulates the basic metabolism of the human body [72]. Previous research has demonstrated that intake of too much or too little iodine can cause the thyroid gland to fail to work properly, resulting in varying degrees of metabolic dysfunction, which affects human health [73]. Inadequate iodine intake can lead to goiter, which is an enlargement of the neck, along with a reduction in thyroid hormone levels, which can damage the brain’s central nervous system. For children, it will lead to intellectual disability and slow reaction, affecting normal growth and development [74]. At the same time, insufficient iodine intake can lead to a decrease in thyroid hormone synthesis and secretion, leading to a decrease in human metabolism, especially in women [75,76]. This disease is caused by the insufficient thyroid hormone in the blood and the metabolism slowing down in the body, named hypothyroidism [77].

By contrast, excessive iodine intake can also harm human health. Several scholars in South Korea analyzed data from the 2017 Korean National Health and Nutrition Examination Survey concluded that excessive iodine intake could also lead to an increased prevalence of hypothyroidism [78]. In addition, consumers exposed to excessive amounts of iodine may be at increased risk of thyroid cancer [79,80]. In general, adults need at least 70 μg of iodine a day and the recommended intake is 150 micrograms per day [81]. However, the study, which looked at seaweed and seaweed foods in the UK, found that eating six products could lead to iodine intake exceeding the limit of 600 micrograms a day [82], the tolerable upper intake levels (UL) for European adults. Meanwhile, nutritionists found that eating just 4 g of dried seaweed reached the maximum tolerance level for adults [72]. Therefore, it is suggested to strictly control consumers’ consumption of seaweed, monitor the total daily iodine intake of citizens, and set corresponding health risk alerts in coastal areas. On the other hand, in iodine-deficient mountainous areas or inland areas, it is necessary to increase food supply from algae, and use algae and its products as raw materials to produce and process iodized salt for sale.

At present, research on the detection of chemical factors affecting the food safety of algae is updated constantly. Iodine in algae is volatile and easily reduced or oxidized, so it is challenging to determine the iodine content in seaweed food. The previous study has shown that the spectrophotometric method can quickly and effectively analyze the iodine content in algae samples [83]. Inductively coupled plasma atomic emission spectrometry (ICP-AES) and inductively coupled plasma mass spectrometry (ICP-MS) have been used to determine iodine concentrations [84,85]. Gas chromatography-electron capture detector (GC-ECD) detection is commonly used and has a detection limit of 0.5 mg/kg [86] (Table 1). However, the consumable reagents, such as pentafluoro derivatization reagents, are quite expensive.

### 4.2. Heavy Metals

In the context of heavy metal pollution, one cannot fail to mention the incident in Minamata City, Japan, in 1956, where the disease was known as Minamata disease. The problem was caused by raw sewage water discharged from factories into the sea, which poisoned fish, shellfish, and shrimp. Consumers ate the seafood and got mercury poisoning, which caused extreme pain in human bones [87]. Therefore, algae, as well as seafood, needs to strengthen the monitoring of heavy metal content. Hwang et al. and Smith et al. studied edible seaweeds in South Korea and New Zealand, respectively [88,89]. They aimed to detect mercury, lead, cadmium, and total arsenic contents in edible algae. As a result, they used these data to alert the public and provide useful information to government departments.

Algae can absorb large amounts of heavy metals, such as mercury, cadmium, lead, copper, and thallium [90]. Methylmercury, as an organic compound, can cause chronic toxic reactions after entering the human body, which may lead to great pain or organ failure [91]. Therefore, this issue has aroused great attention from countries all over the world. Filippini studied the heavy metals and potential risks of seaweed products in the Italian market and proposed that the labels of seaweed food in the market should contain the detection results of heavy metals [92]. In terms of government, the authority needs to set safety limits through legislation.

Using HPLC-MS to determine arsenic content in seaweed products is currently a common and efficient method. Due to the toxic effects of organic and inorganic arsenic on the human bodies are different [93]. As a result, the next phase of research is focused on improving the detection speed and detection accuracy to identify between organic and inorganic arsenic. In order to determine the form of arsenic in algae food analysis, a new method named electrospray mass spectrometry was used by Wiktor Lorenc in 2020 [94]. Furthermore, five Japanese scientists used LC-ICP-MS to measure the arsenic form in seaweed food by LC-ICP-MS and concluded that the inorganic arsenic content in dried seaweed products was significantly increased in brown algae, red algae, and hijiki [95]. Overall, the government and relevant departments should set up limits for arsenic levels in algal foods, as well as consumers needing to improve their awareness of the high risk of seaweeds, containing excessive amounts of inorganic arsenic.

In terms of algal food safety testing technology, the measurement of mercury content in algae food from the original chemical method to the present rapid determination, for example, a simple and rapid detection kit for the toxicity of heavy metal-polluted water [96] and a method for the rapid determination of mercury content in spirulina health food, were established (Table 2). Therefore, food manufacturing industries need to observe changes in mercury content in algal food during different preparation, processing, and preservation processes, in order to better evaluate and control mercury levels in seaweed products.

### 4.3. Sulfur Dioxide

To date, studies have shown that there are two main sources of sulfur dioxide in algae. One is the algae in the growth environment adsorption sulfate and other sulfur substances, but the content is small [99]. The other is adding sulfur dioxide to algal food in food production and processing. In order to prevent seaweed products become oxidation browning or microbial contamination in the storage and processing process, the food processing plants fumigate sulfur dioxide as a colorant and preservative to reach this goal. It has been noted that sulfur dioxide is allowed to be used as a type of food additive in food processing, with color protection, bleaching, anti-corrosion, and anti-oxidation effects. Eating seaweeds with excessive amounts of sulfur dioxide can accumulate in asthmatics and sensitive people, causing serious harm to their health. Some studies have indicated that excessive consumption of SO_2_-treated foods can irritate the mucosal systems of the respiratory tract and lungs, and may be accompanied by headaches, eye inflammation, vomiting, and diarrhea [100]. The most serious problem is that excessive sulfur dioxide in the body will induce the production of cancer cells, a huge potential threat to human health [101]. Codex Alimentarius Commission (CAC) and the United States Food and Drug Administration (FDA) have clearly issued corresponding laws and regulations regarding the additional limit of sulfur dioxide in food [102], in which the limit set by JEFA is not more than 0.7 mg/kg [103].

To reduce sulfur dioxide from seaweeds, the first consideration is to reduce the absorption of sulfur dioxide by algae in the external environment; as for desulphurization technology, these methods are generally adopted (ion exchange, chemical deoxidation, irradiation, and biological conversion) [104]. However, the main factor affecting the food safety of algae is the amount of sulfur dioxide. Therefore, algae food production enterprises should strictly abide by the relevant standards and regulations, the use of sulfur-containing food additives should not exceed the scope and limit, and the browning of algae products and the pollution and reproduction of harmful microorganisms should be controlled through updating processes and new technologies to reduce the consumption of sulfur dioxide as much as possible on the premise of achieving the desired effect. On the other hand, consumers need to emphasize the excessive consumption of sulfur dioxide food health hazards and pay close attention to sulfur dioxide levels in seaweeds to monitor health risk.

To detect the contamination on sulfur dioxide on seaweed, there are three methods to detect sulfur dioxide residues in food: colorimetry, titration, and chromatography in the current stage. In recent years, three new detection techniques have emerged for sulfur dioxide in seaweed products. The first one is a highly sensitive fluorescent probe, which has proven to be very effective in detecting sulfur dioxide derivatives because of its unique selectivity to these chemical substances [105]. In addition, the total sulfur dioxide in algae food was determined by miniaturized dielectric barrier discharge—molecular emission spectrometry, this method has a good linear relationship, accurate detection results, low cost, compact and small detection equipment [106]. Furthermore, a recent study used pre-column derivatization to improve the sensitivity of liquid chromatography for the determination of sulfur dioxide in foods [107] (Table 3).

### 4.4. Pesticide Residue

Pesticide residue refers to pesticides applied to crops, some of which are attached to crops and some of which are scattered in soil, air, and water, where seaweeds grow. Therefore, some pesticides in the residual environment will be absorbed by algae, which will be enriched through direct consumption or food chain, and will enter the human body, because most pesticides are fat soluble [108], such as organophosphorus pesticides and organochlorine pesticides, and will accumulate in the fat in the human body. Resulting in neurotoxic symptoms and even death. In another study, Lorenzo R et al. (2012) used high-performance liquid chromatography-mass spectrometry (HPLC-MS) to analyze pesticide content and the type of pesticide residues, such as azophos, lufenuron, teflubenzuron, and propoxur, have been detected in algae food [109].

This study has led to a growing number of researchers focusing on algae growing in wastewater and its dangers. At present, there are many studies on wastewater treatment using microalgae [110,111], but there are few researches on algae growing in wastewater environment and being processed into food. Although the dangers of industrial wastewater are well known, the hazards of agricultural wastewater cannot be ignored. Some farmers use a lot of pesticides and insecticides to increase crop yields and ensure crop yields. According to research on alpha-cypermethrin, the lipophilic pyrethroid pesticide toxicity of *chlorella*, it is found that toxic reaction to the human body than other pesticides (nausea, vomiting, dizziness), exhibits acute and chronic toxicity, causes insomnia and mental disorders, and may cause DNA mutations [112]. Therefore, this genetic toxicity poses a serious threat to human health. To solve the problem of pesticide residues, various countries have formulated the corresponding maximum residue limit (MRL) standards for pesticides; however, the samples for pesticide residue detection are trace amounts, so improving the sensitivity and detection limit of detection instruments and equipment has become the focus of researchers [113].

### 4.5. Veterinary Drug Residue

According to the reports from 2013 to 2020, some evidence has indicated that antibiotics, antimicrobials, and pesticides are widely used during the period of the breeding process. Xu Dongmei et al. studied the toxic effects of antibiotics (tetracycline and its degradation products) on freshwater green algae, which are frequently used in aquacultural processes [114]. In another research, Joao Rosa et al. studied the potential risks of food safety in aquaculture systems [115]. Both results show that the drugs used in aquaculture eventually end up in the aquaculture environment, leading to the problem of veterinary drug residues in the produced algae food. To avoid a large number of veterinary drug residues in aquatic products, agricultural management departments of various countries require that aquaculture farms are prohibited from discharging untreated sewage.

## 5. Biological Factors Affecting Food Safety in Algae

### 5.1. Pathogenic Bacteria

In general, pathogenic bacteria should not be detected in food. Microbiological testing of algal foods is not only for colony count, coliform, and mold (ready-to-eat dried algae products) but also for *Salmonella enterica*, *Vibrio parahaemolyticus*, *Staphylococcus aureus*, and *Shigella flexneri*. Moreover, the effect of pathogenic bacteria in seaweeds comprises two types of foodborne diseases: infection and poison. In 2016, there was an outbreak of *Salmonella enterica* linked to algae at a local aquaculture farm in Oahu [116]. The main cause of the outbreak has been linked to contaminated seaweed. In another piece of research, Zahid Hayat Mahmud and Afework Kassu conducted isolation and molecular biological analysis of algae food containing *Vibrio parahaemolyticus* [117]. The result indicates that it is urgent to carry out monitoring measures for *vibrio parahaemolyticus* in coastal areas. Hence, it is necessary to strengthen the microbiological detection of algae food and hygiene supervision in the manufacturing environment.

Currently, the traditional method requires the qualitative and quantitative detection of pathogenic microorganisms in algae food through various stages of processing, such as pre-enrichment, selective enrichment, isolation and culture, biochemical identification, and typing [118,119], but this method has limitations, such as cumbersome and time-consuming operation. Therefore, the efficient identification of foodborne microorganisms in algae is the pursuit of scientists and companies. For the detection of foodborne diseases caused by algae, the detection and identification techniques are different, according to the different characteristics of microorganisms. With the development of metagenomic and molecular biology, high-throughput sequencing (HTS) technology is used to read and analyze the DNA of microorganisms associated with food spoilage and foodborne diseases [120]. This technology is used to detect and characterize foodborne pathogens (FBP) in food product [121]. However, there are few HTS operations on algal food. Therefore, it will be more widely used in the microbial detection of algal food in the future. With regard to *Salmonella enterica*, which may exist in the food chain, Bergwerff advocated the addition of immunoglobulin against other invasive microorganisms in the test and believe that the future trend is the combination of more and more microbial detection technologies and nanotechnology, such as immunochromatography, immunosensors, microsphere arrays, and immunomagnetic separation [122]. For the removal, control, and mitigation of these microorganisms that cause foodborne illness, most pathogenic bacteria in algal foods can be thermally sterilized. However, for mycotoxin and some viruses, the product should be discarded when detected.

### 5.2. Algal Toxin

According to the book *The Water Environment: Algal Toxins and Health*, algal toxins are phytotoxins. They are toxic metabolites produced by algae that can accumulate in our food [25]. Early in 2009, Matt Lindon and Steven Heiskary, working at the Minnesota Pollution Control Agency in the United States, measured and analyzed the cyanobacteria toxin in blue-green algae. According to the author, microcystin in blue-green algae is a liver toxin, which can directly bind to the target cell receptors in the human liver [123]. Furthermore, blue-green algae produce a variety of phytotoxins, especially neurotoxins. Researchers have attempted to evaluate the effects of algal toxin; numerous studies demonstrate algal toxins are toxic to human brain nerves [124,125,126]. For example, Aubaeed et al. performed toxicological tests on mice using different doses of toxins produced by blue-green algae, and the obtained results explained that the algal toxin caused significant damage to the liver, kidneys, and reproductive function of the mice. In addition, the toxic damage is not reversible [127].

Recent studies have shown that more efficient and innovative technologies are related to food safety research. According to the survey [128], consumers are most concerned about algal toxins in the seaweeds. As a result, various new testing techniques have been used to detect algal toxins for many years. Natalia Vilarino reviewed the use of photochemical and biosensor or fluorescence microsphere-based determination and other new methods to detect the content of algal toxins in food. By comparing these methods, he found that the method of liquid chromatography-mass spectrometry (LC-MS) with high sensitivity and good detection limit [129]. Thus, with the upgrading of the detection equipment and technology, the traditional method of detecting marine algal toxins will be replaced by efficient instrumental analysis. In terms of toxicological evaluation, using lab mice to do experiments for detecting algal toxins in routine food becomes a way of studying toxicokinetics [130]. Moreover, focusing on rapid detection, Sarah R. Bickman et al. developed a portable biosensor system to solve the problem of rapid detection of cyanobacterial toxins in freshwater [131]. Up to now, electrochemical biosensors have proved to be one of the most attractive analytical devices for the rapid screening of contaminants in food from algae [132]. In another study, some investigators found an approach that has the advantages of high sensitivity, convenience, and effectiveness to detect the content of microcystins from seaweed products, using UPLC-MS/MS and 15 N isotope labelling [133] (Table 4). The research on algal toxins is not only a simple update of detection technology, but also a comprehensive study on the type, toxic effect, and mechanism of algal toxins.

### 5.3. Genetically Modified Seaweeds

With the further development of genetically modified biological engineering technology, the safety of genetically modified food has become the focus of consumers. In terms of seaweed products, genetically modified algae foods have been successfully processed into nutraceutical products [144]. However, three problematic aspects cannot be ignored: the safety of genetically engineered seaweed, potential sensitization, and toxic substances of exogenous genes. Although some researchers showed a positive attitude toward genetically modified algae as a novel food source [145], a recent study expressed a different perspective. This study was done by comparing three different kinds of algae from the genome sequence. At the same time, it also studied allergic reactions by generating new proteins for exogenous genes [146]. As a result, the experimental results showed that such immune allergic reactions caused by transgenic algae would lead to the occurrence of allergic symptoms in consumers, which might endanger their life in serious cases. Another study has demonstrated that the large-scale cultivation of genetically modified microalgae will cause damage to the current ecological environment and increase the risk of horizontal gene transfer through transgenic algae to other organisms in the environment due to the accumulation of the food chain [147].

## 6. The Prospect of Food Safety Research on Algae

As further development of the extraction and research technology of algae active substances, algae are also widely being used in the food industry. The chances of citizens being able to touch seaweed products are increasing, so consumers emphasize the importance of strengthening the monitoring of food safety on seaweeds. Therefore, the demand for algae food safety testing is not only required to develop fast, simple, and low-cost measurement method with high sensitivity and low detection limit of the equipment. It also needs to be connected to new technologies, such as the Internet of Things (IoT) (artificial intelligence, machine learning). Moving to the industry 4.0 era after the outbreak of the pandemic, the IoT is receiving more attention in various industries, including monitoring in real-time and being measured online. Therefore, combining food safety with the IoT is not just a simple update of testing equipment and testing technology. It is a whole process, from the farm (raw materials) to the table chain (products) in the monitoring of physical hazards, chemical hazards, microbial hazards. Eko Ariawan and Stanley A. Makalew address the problem to create a system of sustainable algae spirulina growth monitoring; they constructed a blueprint for a smart micro-farm in 2018 [148]. At the same time, another researcher used the technology of IoT to track food quality and safety in the food supply chain and a mobile app has been successfully developed to detect the freshness of food by using a mobile phone camera [149]. Two years later, Ganjewar also used the IoT to build a food monitoring framework to prevent food spoilage due to changes in environmental conditions during the storage period. It also predicts and analyses the data recorded by sensors to determine the factors affecting food spoilage [150]. This is crucial for the storage and transportation of food from algae.

## 7. Conclusions

Nowadays, algae are mainly used in food, medicine, cosmetics, and industry fields. This review summarizes the three kinds of hazard factors (physical, chemical, and biological) affecting algae food safety and the current situation of seaweed food safety test technology, with a particular focus on the removal, control and mitigation of pathogenic bacteria in algal food that causes foodborne diseases and predicts trends in seaweed food safety testing. Seaweed are rich in nutrients and can be used as a potential resource for anti-cancer, anti-oxidation, and treatment of type II diabetes. Facts have proved that algae foods are not only important food resources in the future but also have great potential in extracting active ingredients and developing functional foods. Therefore, food safety needs the joint effort of government agencies, food processing industries, consumers, and testing agencies. In terms of risk assessment, more attention should be paid to the physical, chemical, and biological risks contained in seaweeds, especially microbiological hazards. In terms of seaweed safety testing techniques, the current safety testing of seaweed foods is mainly focused on compositional studies and ingredient determination [150]. In the current research, these testing methods are designed to detect the safety indicators of algal products. In a developing society, the application of fast, low-cost, and accurate detection technologies and the combination of the Internet of Things (artificial intelligence, machine learning), biosensors, and molecular biology and other new technologies can be combined to achieve simultaneous detection, which is the direction scientists are considering and researching in the future.

## Figures and Tables

**Table 1:** The most common and most advanced technology for the detection of Iodine contaminants.

Testing Item	Technology of Detecting Seaweeds	Advantages	Limitations	References
Iodine	ICP-MS/ICP-AES	High specificity and low detection limit	Pre-treatment is complicated, and the dilution sample easily leads to errors	[84,85]
	GC-ECD	Low detection limit	Consumable reagents are expensive	[86]

**Table 2:** The most common and most advanced technology for the detection of heavy metal contaminants.

Testing Item	Technology of Detecting Seaweeds	Advantages	Limitations	References
Arsenic	Electrospray mass spectrometry	Distinguish organic and inorganic arsenic	Intolerant to complex matrix and high salt	[94,97]
	LC-ICP-MS (Liquid chromatography-inductively coupled plasma mass spectrometry)	High sensitivity, low detection limit, good precision, and wide linear range	Large volume and weight, high price, slow detection speed and high maintenance cost	[95,98]
Mercury	A simple and rapid detection kit	Simple, rapid, low-cost	Only be qualitative, not quantitative	[96]

**Table 3:** The most common and most advanced technology for the detection of sulfur dioxide contaminants.

Testing Item	Technology of Detecting Seaweeds	Advantages	Limitations	References
Sulfur dioxide	Miniaturized dielectric barrier discharge—molecular emission spectrometry	Good linear relationship, accurate detection results, low cost, compact detection equipment	Detection time is long, instrument is complex and expensive	[102]
	Liquid chromatography with pre-column derivatization	Short detection time, high sensitivity, and specificity	Consider using HPLC rather than LC	[107]
	Electrospray mass spectrometry	Distinguish organic and inorganic arsenic	Intolerant to complex matrix and high salt	[94,97]

**Table 4:** The most common and most advanced technology for the detection of algal toxins contaminants.

Testing Item	Technology of Detecting Seaweeds	Advantages	Limitations	References
Algal toxins	Photochemical and biosensor	Smaller sample numbers and shorter response times	Pre-treatment complex, susceptible to environmental	[129,134,135,136]
	Fluorescence microsphere-based	Low cost, simple and low interference, and can detect a variety of toxins	Nonspecific fluorescence limits, sensitivity low sensitivity	[129,137,138]
	Liquid chromatography-mass spectrometry (LC-MS)	High sensitivity, good detection limit, convenience, and effectiveness	Price Instruments are expensive and costly to maintain	[129,139,140]
	Portable biosensor	Portable, rapid, and simple sample preparation	A short service life span	[131,141,142]
	UPLC-MS/MS and 15 N isotope labelling	High analysis speed, high specificity, high sensitivity, high accuracy, high stability	Chromatographic column high pressure, easy to block	[133,143]

## Data Availability

Not applicable.
