# Correction: Wu et al. Current Status and Future Trends in Removal, Control, and Mitigation of Algae Food Safety Risks for Human Consumption. *Molecules* 2022, *27*, 6633

**DOI:** 10.3390/molecules31050839

**Published:** 2026-03-03

**Authors:** Guowei Wu, Dingling Zhuang, Kit Wayne Chew, Tau Chuan Ling, Kuan Shiong Khoo, Dong Van Quyen, Shuying Feng, Pau Loke Show

**Affiliations:** 1 Department of Chemical and Environmental Engineering, Faculty of Science and Engineering, University of Nottingham Malaysia, Semenyih 43500, Malaysia; 2 Institute of Biological Sciences, Faculty of Science, Universiti Malaya, Kuala Lumpur 50603, Malaysia; 3 School of Chemistry, Chemical Engineering and Biotechnology, Nanyang Technological University, 62 Nanyang Drive, Singapore 637459, Singapore; 4 Department of Chemical Engineering and Materials Science, Yuan Ze University, Taoyuan 32003, Taiwan; 5 Institute of Biotechnology, Vietnam Academy of Science and Technology (VAST), Hanoi 100803, Vietnam; 6 Vietnam Academy of Science and Technology, University of Science and Technology of Hanoi, Hanoi 100803, Vietnam; 7 Medical College, Henan University of Chinese Medicine, Zhengzhou 450046, China; 8 Department of Sustainable Engineering, Saveetha School of Engineering, SIMATS, Chennai 602105, India; 9 Zhejiang Provincial Key Laboratory for Subtropical Water Environment and Marine Biological Resources Protection, Wenzhou University, Wenzhou 325035, China

**Removal of References [58,63,108]**

We have removed reference [108] as it was cited as a reference to the main content of the table but does not directly link to the testing item, sulfur dioxide.

**Missing Citation**

In the original publication [1], Desideri, D.; Cantaluppi, C.; Ceccotto, F.; Meli, M.A.; Roselli, C.; Feduzi, L. Radiochemical characterization of algae products commercialized for human consumption. *Health Phys*. **2016**, *111*, 256–264 was not cited. The citation has now been inserted in Section 3.2, Paragraph 2; the last two sentences should read as follows:

According to a study by Desideri et al. [69], human consumption of algae food contains some different concentrations of radiochemical elements. Therefore, seaweed needs to be monitored more closely to prevent radioactive contamination.

In the original publication [1], Aruga, K. *Consumer Reaction, Food Production and the Fukushima Disaster*; Springer International Publishing: Cham, Germany, 2017; ISBN 978-3-319-59848-2 was not cited. The citation has now been inserted in Section 3.2, Paragraph 2; the third sentence should read as follows:

Consumers believe that the radioactive residues of radiation can accumulate in their bodies because humans were at the peak point of the food chain and bioaccumulation [66,67].

With this correction, the order of some of the references has been adjusted accordingly.

**Reference Correction**

In the original publication [1], several references were cited incorrectly or omitted. The authors wish to make the following corrections to the References section:

Replace References [6,18,57,116] with the following:

6. Kim, K.H.; Choi, I.S.; Kim, H.M.; Wi, S.G.; Bae, H.J. Bioethanol production from the nutrient stress-induced microalga *Chlorella vulgaris* by enzymatic hydrolysis and immobilized yeast fermentation. *Bioresour. Technol.* **2014**, *153*, 47–54.
18. Žugčić, T.; Abdelkebir, R.; Barba, F.J.; Rezek-Jambrak, A.; Gálvez, F.; Zamuz, S.; Granato, D.; Lorenzo, J.M. Effects of pulses and microalgal proteins on quality traits of beef patties. *J. Food Sci. Technol.* **2018**, *55*, 4544–4553.
57. U.S. Food and Drug Administration (FDA). *Current Good Manufacturing Practice, Hazard Analysis, and Risk-Based Preventive Controls for Human Food*; 21 C.F.R. § 117.10; FDA: Silver Spring, MD, USA, 2015.
116. Nichols, C.; Ching-Lee, M.; Daquip, C.-L.; Elm, J.; Kamagai, W.; Low, E.; Park, S.Y. Outbreak of salmonellosis associated with seaweed from a local aquaculture farm-Oahu, 2016. In Proceedings of the CSTE Annual Conference, Boise, ID, USA, 4–8 June 2017.

**Text Correction**

In the original publication [1], there was a mistake in the sixth sentence of the Introduction, Paragraph 1. The corrected sentence appears below:

Guiry reported in 2012 that estimates of the total number of algal species ranged widely, from 30,000 to over 1 million. He also mentioned a seminar presentation that suggested an extreme estimate of 350 million species, though he did not endorse this figure. Using the online taxonomic database AlgaeBase, he arrived at a much more conservative estimate of approximately 72,500 species [5].

A correction has been made to the second sentence to the fifth sentence in Section 2.1, Paragraph 3:

Two of the most commercially important categories are kelp and hijiki, though they belong to different taxonomic groups. “Kelp” is a general term often used to describe species within the order Laminariales, particularly the family Laminariaceae. While historically linked to the genus *Laminaria*, commercial kelp also encompasses the genus *Saccharina* [43], as taxonomic revisions have reclassified several species from *Laminaria* to *Saccharina*. For instance, sugar kelp (*Saccharina latissima*) is renowned for its high nutritional value, containing significant amounts of protein, carbohydrates, vitamins, amino acids, and minerals [44].

A correction has been made to the first sentence of Section 2.2:

In meat-processing plants, algal extracts such as hydrocolloids are widely applied as food additives to improve texture and sensory attributes [47].

A correction has been made to the second sentence of Section 3.1, Paragraph 1:

The main reason for this issue is that some employees do not comply with the personnel hygiene requirements of Good Manufacturing Practices (GMP) (e.g., 21 CFR 117.10), leading to the potential inclusion of physical contaminants such as jewelry, hair, plastics, or other foreign materials [25,57].

Two corrections have been made to the third sentence and eighth sentences of Section 3.2, Paragraph 1:

The work highlighted the need for monitoring and management of radioactive contaminants in various sources including algae, to reduce the radioactive cesium values in fish [62].

Some studies have reported that radiocesium, particularly ^137^Cs with a physical half-life of approximately 30 years, is the primary long-term radionuclide of concern released from Fukushima, affecting forest, freshwater, and coastal marine ecosystems [62–64].

Corrections of grammatical mistakes have been made to the first two sentences of Section 5.2, Paragraph 1:

According to the book *The Water Environment: Algal Toxins and Health*, algal toxins are phytotoxins. They are toxic metabolites produced by algae that can accumulate in our food [25].

The authors state that the scientific conclusions are unaffected. This correction was approved by the Academic Editor. The original publication has also been updated.
